# A Rare Case of Primary Breast Osteosarcoma Evaluated with Multiple Modalities

**DOI:** 10.3390/diagnostics11071170

**Published:** 2021-06-28

**Authors:** Tomoyuki Fujioka, Mio Mori, Iichiroh Onishi, Yuka Yashima, Emi Yamaga, Jun Oyama, Kota Yokoyama, Kazunori Kubota, Goshi Oda, Tsuyoshi Nakagawa, Ukihide Tateishi

**Affiliations:** 1 Department of Diagnostic Radiology, Tokyo Medical and Dental University, Tokyo 113-8510, Japan; fjokmrad@tmd.ac.jp (T.F.); 11.ruby.89@gmail.com (Y.Y.); ymgdrnm@tmd.ac.jp (E.Y.); ooymmrad@tmd.ac.jp (J.O.); kota1986ky@yahoo.co.jp (K.Y.); ttisdrnm@tmd.ac.jp (U.T.); 2 Department of Diagnostic Pathology, Tokyo Medical and Dental University, Tokyo 113-8510, Japan; iichpth2@tmd.ac.jp; 3 Department of Radiology, Dokkyo Medical University Saitama Medical Center, Saitama 343-8555, Japan; kubotard@dokkyomed.ac.jp; 4 Department of Surgery, Breast Surgery, Tokyo Medical and Dental University, Tokyo 113-8510, Japan; odasrg2@tmd.ac.jp (G.O.); nakagawa.srg2@tmd.ac.jp (T.N.)

**Keywords:** 18F-fluorodeoxyglucose positron-emission tomography, magnetic resonance imaging, mammography, primary breast osteosarcoma, ultrasound

## Abstract

Primary breast osteosarcoma (PBO) is very rare. This report presents a case of POB that was evaluated by multiple modalities. A woman in her 70s presented with a mass of increasing size in her right breast. A mammogram and an ultrasound visualized a lobulated mass containing coarse calcification in the right breast. Magnetic resonance imaging showed a strong enhancement effect and high signal on diffusion-weighted imaging. Further imaging on 18F-fluorodeoxyglucose positron-emission tomography and computed tomography exhibited a high uptake. A right total mastectomy was performed. Histologic examination revealed abundant periosteal formation, areas of calcification and moderately pleomorphic oval to spindle-shaped stromal cells, leading to the diagnosis of PBO. The presence of calcified breast tumors exhibiting aggressive growth indicates that PBO should be added to the differential diagnosis.

Primary breast osteosarcoma (PBO) is very rare, estimated to account for 12.5% of all breast sarcomas and less than 0.1% of breast cancers. Compared with traditional skeletal osteosarcoma, PBO is more common in older adult patients between age 60–80 years [1,2]. Case reports and small case series have been published in the literature, and cases evaluated with multiple modalities are extremely rare.

A woman in her 70s presented to the authors’ hospital with a mass of increasing size every month in her right breast. She had no significant medical history other than hypertension. In mammography, the right breast showed a 40-mm lobulated mass with relatively smooth borders containing coarse calcification, and the mass was scored as BI-RADS category 4 (Figure 1). Breast ultrasonography likewise revealed a hypoechoic lobulated mass of 40 mm with relatively smooth borders. In power Doppler ultrasonography, the mass contained a punctate-to-coarse hyperechoic area within that corresponded to calcification and blood flow, mainly in the peripheral area of the tumor (Figure 1). Magnetic resonance imaging showed a lobulated mass with strong contrast mainly on the tumor periphery on the contrast-enhanced T1-weighted image (TWI), a high signal in the center of the mass on T2WI and a high signal in the peripheral area on diffusion-weighted images (Figure 2). Additionally seen mainly on the tumor periphery, 18F-fluorodeoxyglucose positron-emission tomography (18F-FDG PET)/computed tomography showed high FDG uptake with a maximum standardized uptake value of 13.7. No other areas of uptake indicated a suspected distant metastasis (Figure 3).

The patient underwent an ultrasound-guided core needle biopsy of the tumor. She was diagnosed with malignant pleomorphic neoplasm of the breast. The patient then underwent a right total mastectomy. Gross examination of the specimen showed a firm mass with a variegated appearance, which measured 5 cm × 4 cm in the upper and the lateral quadrants of the breast. Histologic examination revealed abundant osteoid formation, areas of calcification and moderately pleomorphic oval to spindle-shaped stromal cells. Scattered multinucleated osteoclastic giant cells were also present. Necrosis was found mainly in the central region of the tumor. Multiple sections were sampled, but no epithelial component was found. The patient was treated with successful R0 resection. Immunoreactivity was detected for vimentin but not for AE1/AE3 antibody, monoclonal antibody CAM5.2, smooth muscle α-actin, desmin, DC31, CD34 and the estrogen and progesterone receptors. No overexpression of the HER2/neu oncoprotein was found. The patient was finally diagnosed with PBO (Figure 4).

PBO usually presents as a large, rapidly growing mass [1,2]. The tumor is usually heavily calcified, so dense calcification is seen on the mammogram and ultrasound [3]. A large densely calcified mass is sometimes indistinguishable from a degenerative fibroadenoma [3]. Reflecting the high degree of aggressiveness of this tumor, magnetic resonance imaging typically shows a strong enhancement effect and high signal on diffusion-weighted images, and 18F-FDG PET/computed tomography exhibits a high FDG uptake [4,5]. Although bone scintigraphy was not performed in this case, PBO shows intense uptake on bone scintigraphy [6,7]. The malignant extraskeletal new bone formation is the reason for the strong uptake in PBO [7]. This case highlights the need to add PBO to the differential diagnosis for a patient who presents with a strongly aggressive tumor that increases in size and contains coarse calcification on imaging studies.

## Figures and Tables

**Figure 1:**
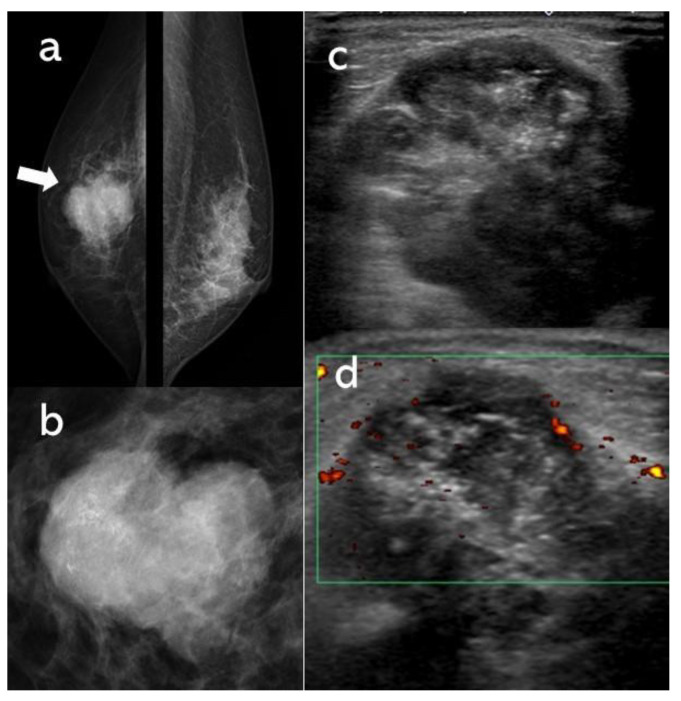
Bilateral mediolateral oblique (**a**: arrow) and right craniocaudal spot compression (**b**) mammograms demonstrate a 40-mm lobulated mass with relatively smooth borders containing coarse calcification. Ultrasound B-mode imaging (**c**) reveals a hypoechoic 40-mm lobulated right breast mass with relatively smooth borders containing a punctate-to-coarse hyperechoic area that corresponds to the calcification and the power Doppler imaging (**d**) showing blood flow mainly in the peripheral area of the tumor.

**Figure 2:**
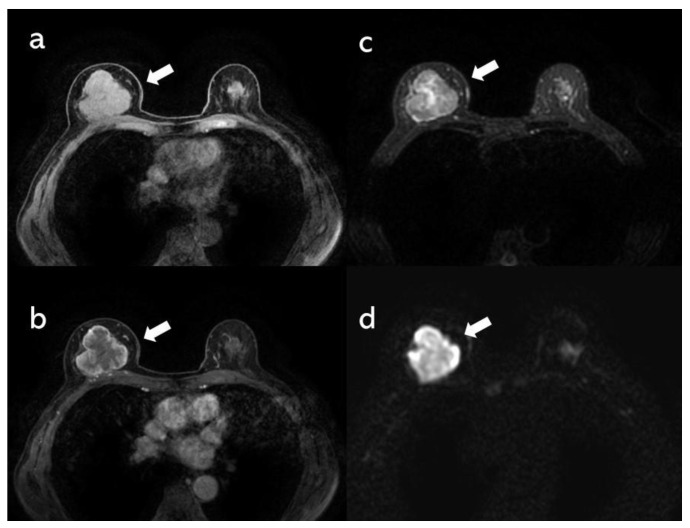
Magnetic resonance imaging shows a lobulated mass on the T1-weighted image (TWI) (**a**: arrow) with strong contrast mainly seen on the periphery on contrast-enhanced T1WI (**b**: arrow), a high signal in the center on T2WI (**c**: arrow) and a high signal in the peripheral on diffusion-weighted images (**d**: arrow).

**Figure 3:**
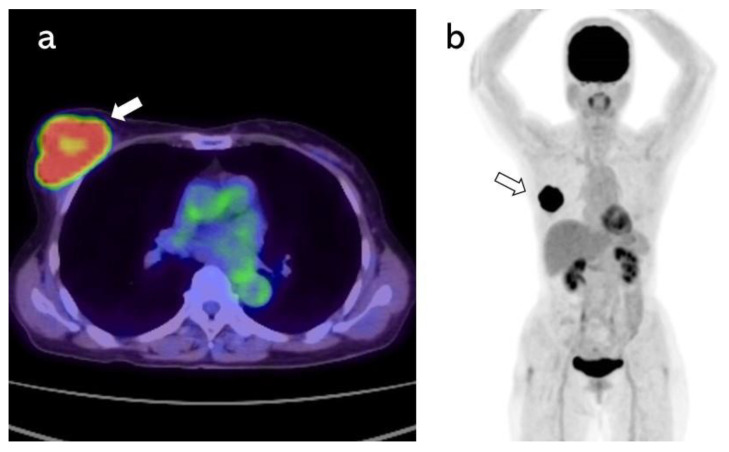
18F-Fluorodeoxyglucose positron-emission tomography/computed tomography shows high uptake mainly on the periphery of the tumor (**a**,**b**: arrow). No other areas of uptake indicated a suspected distant metastasis on whole-body maximum intensity projection imaging (**b**).

**Figure 4:**
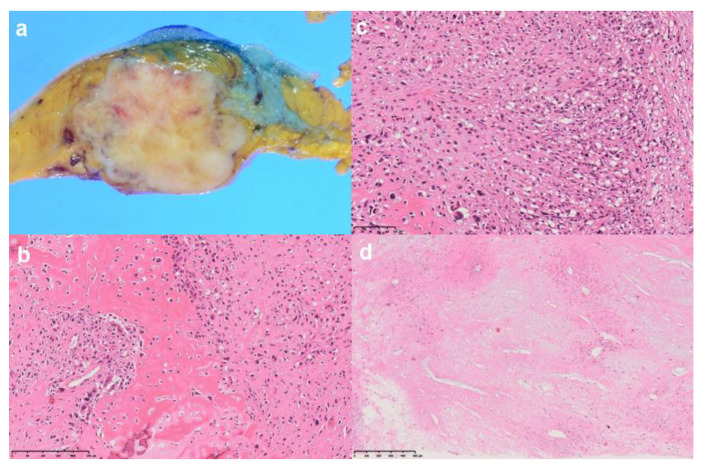
Gross examination of the specimen shows a firm mass with a variegated appearance, which measured 5 cm × 4 cm (**a**). Histologic examination reveals abundant periosteal formation, areas of calcification (**b**) and moderately pleomorphic oval to spindle-shaped stromal cells (**c**). Scattered multinucleated osteoclastic giant cells are also present (**b**,**c**). Necrosis is evident mainly in the central region of the tumor (**d**). The patient was finally diagnosed with primary breast osteosarcoma.
